# Students’ Emotional Experience in Physical Education—A Qualitative Study for New Theoretical Insights

**DOI:** 10.3390/sports7010010

**Published:** 2019-01-03

**Authors:** Sascha Leisterer, Darko Jekauc

**Affiliations:** 1 Department of Sport Science, Humboldt-Universität zu Berlin, 10115 Berlin, Germany; 2 Department of Sport Science, Karlsruhe Institute of Technology, 76131 Karlsruhe, Germany; darko.jekauc@kit.edu

**Keywords:** emotional experience, emotional triggers, student’s perspective, self-determination theory, lifespan physical activity, sport psychology, sport pedagogy, PE teacher

## Abstract

Physical education (PE) can be the starting point for many students to be physically active throughout their lives. Positive emotional experiences in PE are discussed as beneficial for long-term physical activity, however, triggers of students’ emotions are still unclear. The purpose of this study is to explore, from a student’s perspective, emotions and their triggers, which occur in PE classes. N = 12 students (male: six, female: six, ø-age: 15.6 ± 1.2 years) have been interviewed using a focused semi-structured interview to identify their emotions in PE and to explore the situations in which they occurred. An inductive approach with elements of the Grounded Theory Method was implemented to analyze the data. Students reported a wide range of positive and negative emotions. Furthermore, four crucial triggers were identified: (I) Attractiveness of the task, (II) social belonging, (III) competence and (IV) autonomy. Parallels to existing theories, especially the Self-Determination Theory (SDT), will be discussed. These results can be used to improve teachers’ knowledge about students’ emotions in PE in order to build a basis for lifelong physical activity.

## 1. Introduction

The International Charter of Physical Education (PE), Physical Activity, and Sport of the UNESCO states the following in article four:

“Physical education…must inspire lifelong participation… Early positive experiences of play, games and physical activities should be prioritized for all so as to lay a foundation of the knowledge, skills, attitudes and motivation necessary for the maintenance of lifelong participation in physical activity and sport” [1] (pp. 4).

Physical activity provides many physical, psychological, and social benefits for individuals [2]. Therefore, it is important to create abilities and a fundamental motivation for lifelong participation in physical activities from early years on. To support this development, organized youth sport has been in focus in the last years [2]. Consequently, in the context of educational programs for children and adolescents, priority is given to positive emotional experiences to foster the development of a lifelong participation in physical activity [1]. However, PE as a setting of physical activity barely focuses on the opportunity to create a lifelong participation in physical activity, although every child or adolescent participates in PE. The study presented in this article focuses the emotional experiences students have and the triggers of these experiences in secondary PE. It is still unclear if there are unknown aspects of students’ emotional experiences that might provide information about how to create positive emotions in students. Thus, a qualitative approach is used to identify emotional experiences and triggers out of a student’s perspective. As far as the authors know, this study is the first one investigating secondary school students’ experiences in PE by using a qualitative method to focus a student’s inner perspective on PE. Furthermore, this knowledge can help teachers understand students’ emotional experiences and enable every student to have positive emotions in PE.

The difficulty for teachers is to address students’ emotional experiences properly as emotional perceptions change in the course of a student’s school career depending on the development from childhood to adolescence. Consequently, this development influences sporting commitment and related positive experiences of adolescents [3]. Every student gains experiences in PE that can be both positive and negative, for example related directly to situations of winning or losing [4]. However, it is important to build a basis of positive experiences in PE to support students with their sport commitment, although the mechanisms of this affective domain are still unclear. Thus, it is hard for teachers to take students’ emotions into account [4]. The purpose of this study was to investigate students’ emotional experiences in PE. Therefore, this paper reports a qualitative interview study, focusing students’ emotions in PE as a starting point.

Emotion is an important aspect of motivation, behavior, and commitment in PE [3], which helps to foster a lifelong participation in physical activity. Pleasant feelings concerning a specific task makes it worthwhile to turn one’s attention to it, to engage oneself in this task, and to want to do it again. Unpleasant feelings towards a task, however, encourage oneself to avoid the task. Playing basketball in PE is a moment of enjoyment for those students who obviously like playing games, whereas the other students, who do not like playing games at all, try to find a reason for not playing basketball. The last mentioned group shows behavior like asking the teacher for an alternative exercise, or they suddenly feel sick. Unfortunately, these affective aspects are mainly ignored to understand students’ behavior in PE classes throughout adolescence. Additionally, it is important to take into account that emotional experiences change and develop throughout adolescence [3]. To understand the construction of emotions, the structure of emotions can be explained in the circumplex model of affect as dimensional constructions of pleasant or unpleasant feelings with an activating or deactivating arousal [5]. This knowledge about emotions can help to identify students’ emotional perceptions. It is important to help adolescent students develop their emotional intelligence during high school [6]. This means to learn how to release, control, and regulate their emotions, thanks to the development of integrating cognitive functions and emotional perceptions [7].

From a theoretical perspective, there are three main assumptions that were related to affective outcomes: Students’ attitudes towards PE [8], the basic need theory [9], and the motivational climate [10]. Firstly, students’ attitudes are concepts of a cognitive and an affective component of opinions toward a specific content [8], for example, a specific activity in PE like soccer. The investigation of attitudes towards PE shows that students who find a personal relevance in PE have positive affective outcomes, for example, enjoyment in soccer [11,12]. It seems as if students who participate in sports during their leisure time perceive more positive emotions in secondary school PE due to their positive attitudes toward sports in leisure time [12]. The emphasis on competitive sports in secondary school PE seems to affect students’ emotions in PE positively, when they do sports in their free-time, or negative, when they are more inactive [13]. Secondly, the basic need theory [9] describes that fulfilling the needs for autonomy, relatedness, and competence supports a self-determined motivation that is in association with positive emotions [14]. Fulfilling basic needs in a PE situation where students experience positive emotions helps to motivate students intrinsically [14,15]. The investigation of social relatedness shows that social interaction in secondary school PE is associated with positive affective outcomes [16]. Autonomy-supportive teaching styles provide the possibility to fulfill the basic need of autonomy and is thought to be related directly to positive emotions [17]. Competence seems to be positive related to need satisfaction and thereby positive affective outcomes too [18]. Thirdly, motivational climates create learning atmospheres. An ego-oriented motivational climate in PE results in a competitive environment, whereas a task-oriented motivational climate creates a learning atmosphere focusing on the process of learning [10]. Motivational climates have been investigated in relation to students’ emotions in PE [19,20,21]. A task-oriented climate in class supports positive affective outcomes for students in PE [19,21]. While investigations of different motivational climates as task versus ego-orientation provides information about affective outcomes in PE, it still remains unclear if there are any other possibilities to create a fun-related learning atmosphere in class [22]. Finally, applying theories, like students’ attitudes, the basic need theory or the assumption of motivational climates leads to affective outcomes in secondary school PE. However, an evidence-based description of distinct emotions and their specific triggers in secondary school PE is still missing.

This theoretical perspective on emotional outcomes represents a specific view on which theories are related to emotional outcomes in PE, but does not identify distinct emotional experiences of students. Current literature focused more on affective outcomes mainly categorized as positive or negative. The question remains, whether or not research misses the investigation students’ distinct emotions and their specific triggers in secondary school PE. In fact, there is no research that focuses a student’s perspective to prove, if there are unrevealed aspects of emotional experiences in secondary school PE. In contrast to a hypothesis testing [23], a qualitative methodology can explore a student’s perspective to derive new theoretical assumptions [24]. As shown in an investigation of primary school students [25], it is possible to investigate emotional experiences and its triggers in primary school PE by interviewing students. A first exploration of emotional experiences is presented in a qualitative interview study [25] that analyzed students’ emotions in primary school. The results of this study show that younger students have broad emotional experiences that are mostly related to basic pleasant or unpleasant feelings to motor tasks, for example, fun in trying risky tasks or anger if they do not succeed [25]. However, the question what changes in the emotional experiences can be detected when the students get older and visit secondary school arises. Thus, it is important to gain insight into students’ emotional experiences at secondary schools. A similar study in secondary school PE, which focuses on what emotionally changes during the transition from childhood to adulthood, is still missing, in contrast to primary school students where there have been studies in the past. If we gain a more distinct insight in what adolescent students experience in PE and how these experiences are triggered, we would be able to focus on how to support students in regulating their emotions.

Thus, it is necessary to explore the emotional experiences out of a student’s perspective. An explorative interview study could provide information about unrevealed aspects of student’s emotions in PE. In sum, it remains unclear what exactly students experience emotionally in secondary school PE and how these experiences are connected to individual and environmental triggers. In order to fill this research gap, we ask the two explorative research questions: (1) Which emotions do students experience in PE, and (2) what triggers students’ emotional experience in secondary school PE?

## 2. Materials and Methods

To answer the research questions, a qualitative focused semi-structured interview study with an inductive strategy was conducted in order to explore the field of emotional experiences in PE classes from a student’s perspective. Students who participated in German secondary school PE classes were interviewed. The Public Administration for Education, Youth, and Science in the urban region where the study conducted was approved the ethical reasonableness of the entire study for the students. The study adhered to the data policy valid at the time when the study was conducted. Collecting the students’ names or any other pseudonyms to identify the participants were restricted by the Public Administration for Education, Youth, and Science.

### 2.1. Study Design

Twelve ninth and tenth graders participated, six boys and six girls, aged 14 to 18. The average age was 15.6 years. Half of the participants were physically active outside of school. The other half only took part in the PE classes provided by their schools. The teachers confirmed that the same pedagogical framework was used for all the participants’ PE classes. All students were chosen randomly and participated voluntarily. No compensation was given for participating in this study.

As a first step to creating the interview structure, questions relevant to the topic were brainstormed, discussed, and selected with and by peer researchers. To avoid overlaying experiences of the interviewer, questions focused on remembering and visualizing real experiences of and by the interviewee. In a second step, questions were put into an order that enabled fluent communication. Subsequently, the interview structure was tested on two students (both female, age: 14) who participated voluntarily. Due to the results of this pilot study, the participants were briefed and it was pointed out that there were no wrong answers. According to our pilot interview partners, this statement relaxed the atmosphere. In order to support the participants in describing their emotions, the interviewer explained before the interview that it might be difficult to name emotions. However, every paraphrased explanation would be as good as a single word for one emotion. This reduced the participants’ struggle trying to find the ‘right words’; they were able to proceed with their individual narrative strategy. Their answers were never overlaid by the questions of the interviewer. All adjustments were made before using the interview structure in the study.

**Figure A1:**
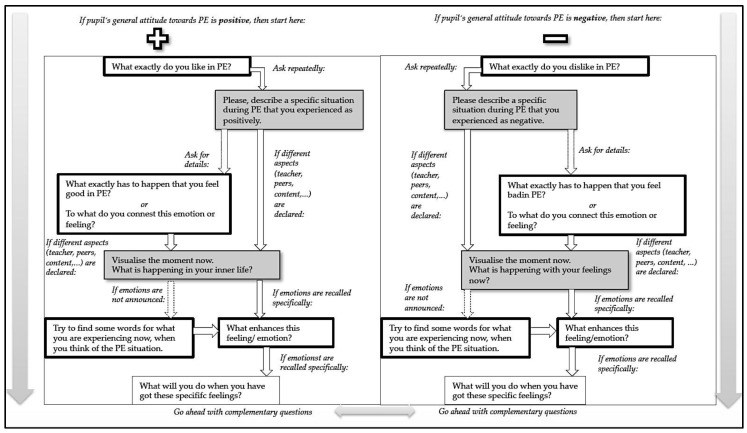
Interview questions and schema of the interview guideline used in the present study.

The interview procedure was standardized in accordance with interlocutors, sequences of the communication and the interview structure. Interviews were carried out on a one (interviewee) to one (interviewer) basis with an average duration of 20 min. The interviewer remained the same. Interviews were carried out in a separate room in schools during regular classes. Students were picked randomly from the on-going PE class. Firstly, a short introduction was given introducing the interviewer and his assistant, informing the participant of the aims and the procedure of the study, as well as their rights as voluntary participants. Secondly, the interviewee was asked introductory questions (e.g., age, favorite subject or free time activities) to start the conversation. Then the interviewee was asked about his or her general attitude towards PE in school. This beginning of the interview was chosen as attitudes are related to emotional experiences. Thus, if the answer to this first question was positive, questions about positive emotional experiences were asked directly afterwards. This would be followed by questions about negative emotional experience. If the answer was negative, questions were asked the other way around. Interview questions were for example, “Please describe a positively/negatively experienced situation during a PE class in which you participated”, “What did you feel in this situation?”, or “What exactly made this situation more or less intense?”. The entire interview guideline is attached in Appendix A, Figure A1. Further ad-hoc questions were asked to clarify the emotional experience and to get more details. To obtain further information and to conduct member checks during the interviews, the interviewer used interrogation techniques of paraphrasing and repeating the interviewees’ answers followed by a question (e.g., “Can you elaborate this please?”).

### 2.2. Data Analysis

For the analysis, the interviews were audio-transcribed using the transcription software F5 [26]. Following the hermeneutical idea of coding, comparing and contrasting in several repetitions with MaxQDA [27], a theory of emotional experience in PE was derived [24]. To establish this theory, the transcribed interviews were first coded openly to identify elements that answered the research question [24]. Secondly, axial coding made it possible to integrate similar codes into dimensional categories of trigger mechanisms as well as influential categories [24]. Inductive and deductive approaches to analyze data were applied [24]. The inductive analysis identified influencing aspects and triggers of emotions in PE situations described in the interviews. During the deductive analysis the circumplex structure of emotion by Russell and Feldman Barrett [5] clustered the emotions named in the interviews. Third and lastly, selective coding evaluated trigger mechanisms and influencing categories concerning the explicit emotions named by the participants. Every step was accompanied by a peer discussion to establish a convincing step-by-step analysis of the data.

## 3. Results

**Figure 1:**
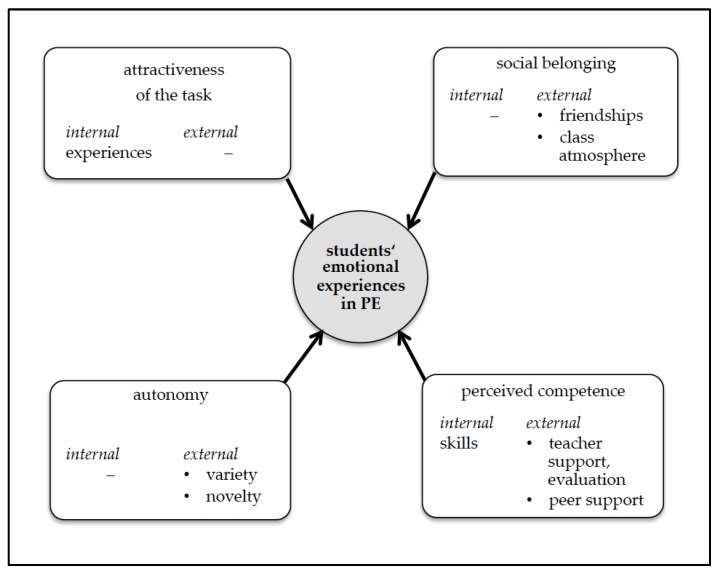
This is the model of a student’s emotional perception in physical education (PE). The emotional perception can be triggered by the attractiveness of the task, social belonging, perceived competence, or autonomy.

We derived the model presented in Figure 1 from interviews with students. Four main categories of emotional triggers were identified: (I) Attractiveness of the task, (II) social belonging, (III) perceived competence, and (IV) autonomy. The following section explains every context of a trigger. For each of the four triggers, we will first give a description of the trigger. In the second part, we will explain the influencing factors of the trigger. Finally, we will summarize the emotions identified and triggered by these factors. Students’ names are pseudonyms.

### 3.1. Students’ Emotional Experiences

In conclusion, a wide range of different emotions both on the positive and negative spectrum of students’ emotional experiences in secondary PE can be identified in the interviews. An overview of all identified emotions, categorized into positive and negative emotions, is given here. In the following sections, the emotional experiences are categorized additionally in activating and deactivating according to the circumplex model of affect [28].

On the one hand, there are positive emotions and related states, such as happiness, joy, fun, rush of adrenaline, passion, contentedness, freedom, balance, compensation, relief, relaxation, pride, confidence, skilfulness, grandeur, power, superiority, enjoyment, sense of aesthetics, and tension.

On the other hand, there are negative emotions and related states, such as depression, grief, weakness, helplessness, feeling cheated, fear, disappointment, discontentment, (negative) astonishment, embarrassment, humiliation, rage, aggression, anger, animosity, and boredom.

### 3.2. Attractiveness of the Task

Interviews show that attractiveness of the task describes a student’s interest in a specific motor task. It is an internal task-specific trigger that can be learned. This means, the attractiveness of the task depends on motor experiences formed during previous physical activities. These experiences are internal representations of motor tasks that the students had to perform in the past. Hanna, for example, likes to practice on a beam. She says: “…if you make it—it is simply this…small rush of adrenaline” (Interview-3). Former experiences influence the attractiveness of the task as an emotional trigger. Only if students remember previous positive individual experiences connected to the specific task or see similarities in this task with a known and liked task, then this is a positive trigger. Linda’s experiences as a cheerleader enable her to include motor experiences that she enjoys in this specific PE-task. This, too, helps to foster her commitment to PE because she can incorporate what she likes from cheerleading into gymnastics. Finally, she concludes that she enjoys investing her time and skills into the task. When we asked Linda to describe this joyful experience, she explains:

“I like special tasks. In winter, we did gymnastics and fitness. This was very fun, and when we started, … I thought: “Finally, we can do it again!” It’s been a year since we last practiced it. We do it every year, and I can hardly wait for gymnastics. When we start again, I am so excited… It is always so much fun because we can perform a lot. Since I have been a cheerleader for five years, I can show a lot more than expected, things we learned in cheerleading like lifts and entertainment…” (Interview-7).

Compared to Linda, Anna shows that individual experiences—“this is not my sport”, as she says (Interview-10)—influence the attractiveness of the task (Anna does not like games in PE) in a way that finally triggers negative emotions such as a lack of interest or boredom. Anna, 15 years old, makes clear that she is absolutely not interested in games: “… In general I am not that kind of girl who likes games, this is not my kind of sport…” (Interview-10). Whenever she has to play in PE, she feels uncomfortable and dislikes this specific PE lesson.

From the data, we summarize that on the one hand the attractiveness of the task triggers positive emotions that are pleasant and highly activating, for example, excitement or joy, and on the other hand, negative emotions that are unpleasant and barely activating, for example, boredom. We can conclude that the attractiveness of the task is, as in Linda’s example, an important predictor of positive emotions in PE, like feeling “joyful”, “happy”, and “excited”. Anna’s example shows the opposite reaction to the teacher’s announcement of playing in the following PE lesson.

These stories are good examples for the relationship between attractiveness of a specific task and the perception of positive and negative emotions. Past motor experiences play a crucial role and are an influencing aspect in perceiving a task as attractive.

### 3.3. Social Belonging

The perception of social belonging is represented in the interviews by a perception of interaction among peers. Selma, 15 years old, is not very interested in participating in sports but whenever the entire class is involved she has “fun” during the PE classes:

“Our fun lessons are the game lessons when we play together… And we are not so ambitious to win; we just want to have a good time together. We are all friends, and we know each other pretty well. That is how I see it… if I had to name it in one word, it is about belonging. Everyone is part of it, and everyone is accepted. And this makes you feel happy, it is fun, and you do not play against the others, you play with them” (Interview-2).

In contrast to this, Peter, 14 years old, shares an intensely negative experience with us when he remembers a group exercise with low cooperation and social commitment. Peter’s words show that he had expected collaboration during this group exercise. When he describes that quitting the group would have made his experience worse, we can interpret this as downgrading the feeling of belongingness: Losing the group means losing the sense of belonging:

“… we had to perform a gymnastic routine in a group building human pyramids; I am normally the guy at the base. The others, though, did not want to climb up. They just said: “Nah, this is not what I fancy doing!” And our teacher had already said that this is not okay. Then, I felt betrayed! They just gave up in the last rehearsal. I just did not understand how this cooperation and team spirit could not exist anymore. That I just could not rely on the others, it was the uncertainty, this feeling of “why are they doing this?” I really did not understand why they gave up. And this was a mixture of sadness and anger, and I was in total rage because of them. And I felt down and depressed” (Interview-1).

External factors like friendships between classmates or a good social atmosphere in class influences the perception of social belonging. Social belonging is characterized by a degree of social cohesion and engagement, motivation, inclusion or exclusion, and admiration or rejection. Whereas social cohesion and engagement is the perception and concrete action of interpersonal dependence, as Leonard describes: “I devoted myself to the other guy, because I realized that he was active on my behalf, he would even make a sacrifice for me in the match by falling out of the match” (Interview-4); motivation means to push on with the support from another peer group member, like in Jack’s experience: “…the others [students] who have already finished the exercise but still run with me to motivate me, they really go along with me I improve my results and feel really good” (Interview-8). Inclusion or exclusion defines interpersonal behavior above average or the general perception of being part of a certain community like being with friends as Peter puts it: “Well these are the people I trust most, and otherwise it would not be as much fun, my friends do have to be there” (Interview-1) or like watching other students that distance themselves from the others as Marie says: “I do not like the guys …who are sitting on the bench, who are doing nothing but talking, who do not help. I think this is impolite” (Interview-9).

Data shows that social belonging mainly triggers positive emotions with pleasant and slightly activating characteristics like fun or happiness. However, a lack of social belonging seems to lead to negative emotional experiences of unpleasant and slightly deactivating (for example sadness) or slightly activating characteristics (for example anger). Selma describes her feelings as positive when playing together. Peter’s story shows that a reduction, loss or lack of social belongingness triggers negative emotions in PE and that this atmosphere influences the type of negative emotions.

The interview examples show that social inclusion triggers the emotional perception. However, it is also obvious that social belonging does not exist on its own. This feeling of being “part of it” depends on the atmosphere and friendly relationships in class.

### 3.4. Perceived Competence

Interviewees described competence as perceived success in motor tasks, like learning or improving in an exercise or achieving personal goals (e.g., success by overcoming fear) or competitive goals (e.g., achievement in comparison with others) in sports. Perceived competence becomes evident in the interviews of Jack, Marie, and Selma. Soccer player Jack, 15 years old, describes success in a soccer game, and Marie, 16 years old, explains how she succeeded on the horizontal bar. Both Jack and Marie describe situations in which they succeeded. Jack succeeded in scoring a goal, whereas Marie learnt a specific move in gymnastics. Jack says:

“… I kicked the ball and directly scored a goal… It was a positive feeling that I had made it. And the others sometimes tell me: “What you did was really cool.” This positive feedback is a good thing for me. I feel better in soccer when I know that I did it right. And it is fun; playing a game you are good at. … I feel certain when I know that I can succeed in my performance” (Interview-8).

Additionally, Marie explains:

“Recently, we practiced at the horizontal bar. I did not make it. But my teacher helped me, explained and showed me how to do it. And then I learned it and finally succeeded. And this success felt good. … and my teacher told me that it was very good… Finally, I was really happy and relieved!” (Interview-9).

Perceived competence depends on external or internal factors. External factors are environmental aspects that influence the perception of competence due to feedback, evaluation, help, or support by others: “It was good to know that my teacher trusted me, and that she knew I could succeed. This boosted my will to succeed. I wanted to make it. My teacher supported me, and this was my motivation”, says Marie (Interview-9). In contrast, internal factors are students’ inner principles that affect their expectations and perceptions of being competent, like individual values, ambitions, or the will to succeed. Harry explains these inner principles referring to his ambitions: “Actually, I have my goal in mind… to be faster [when running] than before. This only works out when I am very, very motivated, when I do not give up, and when I am very ambitious” (Interview-6). There are internal influential aspects, as well. Jack is a good soccer player in his free time, so he seems to be fit, and his motor skills for the match are well developed which makes him feel certain (“I feel certain when I know that I can succeed in my performance”). Marie mentions her “will to succeed”, so she refers to a mental skill that helped her in gymnastics. Thus, we suggest that individual skills are either physically or mentally important influential factors on being able to perceive competence as a trigger of positive emotions. Jack would not have been as successful as a soccer player without his skills and experiences, and Marie would have stopped trying to learn this specific move in gymnastics if it had not been for her strong will. Even bad motor experiences in the past can influence the perception of competence, but the emotional perception however is negative. Selma, for example, is not very interested in sports. When we asked her what she does not like in PE, she answered rapidly: “Cross country running, I am so bad at cross country running”. Every time she has to run, she feels incompetent. Here is her story:

“… I feel really uncomfortable when I run two laps in eighteen minutes, and even though I feel I was really fast, my teacher tells me that I will still get the worst grade because I was too slow. It was so exhausting. I did my best, but I still got the worst grade. No reward! This is so humiliating; I do not want to carry on because I think that I will never change. It makes me feel disappointed, angry and sad at the same time. And I feel bad because I could not do any better…” (Interview-2).

Positive emotions in this context seem to be very pleasant but barely activating (for example enjoyment or relief), whereas negative emotions seem to be very unpleasant and slightly activating (for example anger) or deactivating (for example disappointment). For Marie and Jack, the final motor success triggered positive emotions such as “happiness”, “joy”, “certainty” (Jack: “You know, you did it right”), and “relief” (Marie: “finally succeeded”). Selma’s description contrasts that of Jack and Marie. Feeling incompetent at cross country running makes Selma feel “uncomfortable”, “humiliated”, “angry”, “disappointed” and “sad”.

The stories of Jack, Marie, and Selma show that perceived competence is a trigger of emotions in PE, but this trigger is strongly related to the support from their peers and teachers, the evaluation by others, and their individual skills.

### 3.5. Autonomy

Data has shown that autonomy can be defined as students’ freedom of choice concerning activity or their own behavior. The satisfaction of autonomy can trigger emotions simply by having the right to choose: “…that you choose something [a task] you are good at…” (Interview-2). Additionally, it can influence competence when the student can make his or her own decisions about a task, as Selma explains: “… for example those who are good at soccer, can play soccer, if they want to” (Interview-2). Students can then choose to be active in whatever activity they are good at.

Interviewees explained two distinct external influential aspects of autonomy: variations and new tasks. Variations are, for example, individual changes to a task that students can choose from or alternatives to specific tasks. New tasks are a novelty in relation to the regular curriculum in PE. Yet, they have to be discussed and chosen by the students to support autonomy. Anna gives a clear example of novelty when she explains that she appreciates her teacher being more flexible with the curriculum to make a step towards the class’s interest “… then she [the teacher] makes the most of it. For example, we recently danced which was different from what we normally do” (Interview-10). Both variation and novelty are conditions for supporting autonomy in PE.

The results show positive emotional experiences that are triggered by the satisfaction of autonomy, pleasant, and more activating (for example, excitement when looking forward to the task) than the emotions triggered by the satisfaction of competence. According to Marie, her teacher asks the students to express their wishes and ideas for the PE classes. With this democratic approach, novelty leads to perceived autonomy. Autonomy triggers positive experiences and as Marie states: “this is a great thing to happen in every PE lesson. I am always looking forward to our PE classes” (Interview-9). With our data, we cannot contrast autonomy as a trigger of positive emotions with a negative experience. No interviewee reported negative effects concerning autonomy. Autonomy supportive situations in PE attract students to a specific task. Mainly, variety and novelty influence these situations positively.

## 4. Discussion

The purpose of the present study was to explore from a student’s perspective the emotions of adolescent students in PE at secondary schools, and how they are triggered. The following section, in turn, presents the key findings concerning students’ emotions and integrates the findings of students’ perspectives into a theoretical perspective. Thanks to the interview design, we could determine distinct emotions out of a student’s perspective, in contrast to former research findings that focused more on positive or negative affective outcomes. Secondly, the derived model and its close relation to self-determination theory (SDT) is discussed. In this section, we will emphasize our findings on the attractiveness of the task that can be compared to students’ attitudes and personal relevancies towards PE. Equally we focus on the parallels between our findings and the basic need theory and its relation to positive affective outcomes. Finally, this model’s implications are explained, and the methodology of the study is discussed.

### 4.1. Which Emotions Do Students Experience in PE?

To explore the field of emotions in PE in young people, our first research question was: Which emotions do students experience in PE? The interviews revealed a variety of individual insights into emotional experiences. The identification of a broad range of both positive (happiness, relief, and others) and negative (anger, sadness, and others) emotions assists in the understanding of PE from a student’s perspective. The results show that students experience a broad range of activating emotions in PE, be it pleasant (for example fun, joy, or excitement) or unpleasant (for example anger). Yet, it is also possible that students experience deactivating emotions, which seem to be limited to unpleasant feelings, like sadness or disappointment. Regarding positive emotions and related states, primary school students mentioned being cheerful, happy, contented, joyful, relaxed, and passionate in PE as well as feeling fit, free, and relieved [25]. In comparison, the present study confirms these emotions with secondary school students. This confirmation had been anticipated due to previous studies that describe positive emotional experiences in sports as remaining stable in the transition from childhood to adulthood [3]. Additionally, pride as a self-conscious emotion [29] appears in the presented study’s results. This might be related to a broader sense of self-concept of adolescents in comparison to that experienced by children; adolescents start to be aware of their own actions and how these are seen by others [29]. Thus, pride is an emotion that results from comparing one’s own appraisal of performance to that of others. The present results show that the opinions of peers become more important for emotional experiences in secondary school PE than in primary school PE.

When contrasting the negative emotions and related states identified in this study with previous research, some differences can be seen. Fear, boredom, weakness, grief, depression, rage, and anger have also been identified in the experiences of primary school students [25]. Other negative emotions, identified by our study, such as embarrassment, humiliation, and the feeling of being cheated on, are emotions that develop within the social context of PE over time, since to young people, acceptance by peers is more relevant [3]. In accordance with Harter’s theory about the development of one’s self [29], we can again confirm that the social context in secondary school PE classes plays an important role. For example, in PE there is both a broader range of negative emotions, due to social interaction, and of self-conscious emotions.

### 4.2. What Triggers Students’ Emotions in Secondary School PE?

Our second research question in exploring the emotions of young people in PE was: What triggers students’ emotions in secondary school PE? We identified four triggers of emotional experience: (I) Attractiveness of the task, (II) social belonging, (III) perceived competence, and (IV) autonomy. These triggers are similar to recent findings on how to support adolescent students in finding a meaning in PE through personal relevance [11] or satisfying basic needs [12,13,30,31]. We will discuss these triggers in the following section and will contrast our findings with theories that may provide further knowledge of students’ emotions in PE in order to support students with the development of a lifelong motivation to be physically active.

(I) The attractiveness of the task is a trigger of positive or negative emotions. The present findings show that attractiveness is related to interest or personal relevance [8,11]. According to previous research [11,12], the student perceives positive emotions, like joy, if he or she evaluates the task as attractive. This supports our results. In contrast, low attractiveness results in a lack of interest or boredom during the task. The mechanisms that were identified influences the development of students’ interest and their positive feelings [11,32]. Consequently, this interest leads to commitment to sports [33,34]. Our exploration shows that experiences are an important factor in making a task attractive. The interview partners of our study, for example, remember previous experiences they made in PE or in extracurricular physical activities. According to Weiner [35], expectations are an important aspect of emotional attribution and a predictor of enjoyment [36]. In terms of PCK, it is for teachers to consider that students need to collect different experiences in order to be able to learn what makes a task attractive. Finally, teachers could also consider alternative tasks, which could potentially be more attractive.

(II) Social belonging amongst peers plays a crucial part in PE. To experience positive and negative emotions, the degree of how strongly one feels part of a social group is very important. The word “belonging” even appeared in the interview. These social bonds create emotional bonds that also foster intrinsically motivated behavior [9]. Thus, social bonds are beneficial to PE classes in secondary school. In contrast to the more self-perceptive experiences of primary school students [25], the present findings support the assumption that adolescents are more sensitive to their peers and friends as well as to the atmosphere in class. In accordance with former assumptions [3], our results show that being accepted and involved, mainly by peers, is, for secondary school students, important for triggering emotions in PE. In regression analyses [37], positive social relations in PE are important for positive emotional outcomes. Especially if the relationship between the teacher and the students is not positive, the relationships between peers are discussed as very important to enhance positive affective states in PE [37]. Present findings of a qualitative study highlight peer relations as one important aspect for positive emotional experiences in PE [38]. Thus, we can add our results to this recent discussion. For students, social interaction is an important aspect to perceiving PE as meaningful, which is related to positive emotional experiences too [11,16]. Thus, concerning teachers’ pedagogical work, investing in a good and cooperative social environment in secondary school PE has multiple benefits for students’ emotional experiences and the related aspects of their self-development.

(III) Perceived competence is a strong trigger for emotional experience. The feeling of competence/incompetence in a motor task leads to either positive or negative emotions and depends on one’s skills, either physically or mentally. We assume that perceived competence is strongly related to success when performing tasks in PE. The present results show that internal factors influence the perception of competence, as seen in determination, because one has either met expectations or has the ambition to utilize one’s skills. In this context, the results of this study support the assumptions of the attribution theory [35], which states that knowing one’s own skills from former experiences enhances controllability, which in turn triggers emotions. However, it is not only controllability as an internal factor but also external factors such as social support or evaluation from others that influence the perception of competence. Social support or evaluation by others is what we call an external factor. According to former research findings [33,39], support from either fellow peers or from the teacher, influences young peoples’ perceptions of success in the context of sports. This is similar to what we have seen in the interviews. Finally, evaluation is an important element for feeling competent [35,36], as described in excerpts of the interviews. In turn, evaluation is an important aspect for young students to feel competent and therefore to experience positive emotions. This finding is quite similar to the explanation of positive emotions in the context of SDT [40]. Perceived competence, in terms of physical self-concept, is a significant predictor of future physical activity, which moderates the relationship between motor abilities and physical activity in adolescents and young adults [41]. Furthermore, perceived competence in PE is related to creating meaningful PE classes that are connected with positive emotions [11]. Concerning teachers’ PCK, it is important to acknowledge that supporting students to set and achieve their goals, as well as evaluate their performances comprehensibly is vital in the context of perceived competence as a trigger of emotions.

(IV) Autonomy is a trigger of positive emotions since it relates to students’ freedom of choice and wishes. Students appreciate being able to decide for themselves what they want to do in PE, or decide that they like to try new things, in accordance to their wishes. In a similar qualitative study [38], autonomy is defined in the same way as we derived from our data. In unison with this definition [38], we see autonomy in PE as a construct of free choices, variety, and novelty that is closely related to positive emotional outcomes. This description of autonomy corresponds with the term, “autonomy supportive teaching style”, which is widely used in literature [17,42,43]. This didactical approach supports students’ enjoyment and interests in PE tasks [17,44], and we can see the positive effects in our results. In the context of PCK, teachers should choose a way of teaching supporting autonomy to trigger positive emotions. The results of our study do not support the idea that the lack of autonomy is a trigger for negative emotions. Students may not be aware of a lack of autonomy. Therefore, a lack of autonomy does not necessarily trigger negative emotions. Another explanation might be that students are used in an authoritarian way of teaching and do not expect autonomy.

Triggering students’ emotions by creating an attractive, interactive, competence-oriented, and autonomy-supportive PE can help teachers train the students’ emotional intelligence. Thus, teachers can create situations with knowledge of triggering emotions to provide emotional intelligence training within PE. This work on emotional intelligence contributes to a secondary school PE that is sensitive to the emotional development throughout adolescence [6].

The present findings create a basis to support students by motivating them to be physically active in the long term. In addition to recent research findings on triggers of adults’ emotional perceptions during recreational physical activities [41], the present findings support perceived competence and social belonging as important emotional triggers in physical activities. However, it seems as though autonomy does not play a crucial role in physical activities for adults. However, it is a novelty that triggers these emotions. In contradiction to our findings where novelty is a condition of autonomy, Wienke and Jekauc [41] shows that, for adults, novelty itself is a trigger for physical activity. Here, we probably see a development of a trigger due to the setting. In PE, students depend on a teacher’s planned lesson and his or her way of teaching supporting autonomy, whereas adults may freely choose their physical exercise. Going from PE to adult physical activity, novelty itself becomes the trigger. To support this development, teachers may ask for their students’ opinions so they can integrate new tasks into the lessons. If students learn to choose new tasks they are interested in doing in PE, we suggest that novelty will become an even stronger trigger of positive emotions in physical activities later on. Finally, teaching supporting autonomy in PE seems to be an important element for positive emotional experiences for physical activity in the future.

### 4.3. Present Results in the Context of SDT

The findings of the present study are closely related to SDT, particularly with the three basic needs as emotional triggers: Social belonging, perceived competence, and autonomy [9]. We derived these results from our data, although we did not use the approach of SDT to analyze the interviews. This is in accordance with a recent study [38] that also found close relations between the basic needs and emotional experiences, especially in the context of autonomy and social belonging. According to the literature [9], intrinsic motivation requires the fulfillment of these three needs. Surprisingly, we identified social belonging, perceived competence, and autonomy not as basic needs but as triggers for emotional experiences in PE.

The role of emotions in SDT has already been discussed. Different types of motivation depend on different emotional regulation strategies [9]. Other studies [45,46] add to the discussion that positive affects, which are the prerequisites for positive emotions, are signals for intrinsic motivation according to the SDT. It is supposed that students perceive more positive emotions when they are motivated intrinsically and that there is a reciprocal relationship [40]. Despite this, though, emotions have not yet been integrated into the SDT [45,46].

Regarding the interaction of former experiences and the attractiveness of the task as an emotional trigger, this can confirm the reciprocal relation between intrinsic motivation and emotion. As we did not focus on emotional regulation strategies in this exploration, we cannot compare our findings with the suggestion that these regulation strategies are important [9]. However, we do see parallels to the idea that positive emotions enhance intrinsic motivation. That is why emotions have to be included into the SDT as an influential factor of intrinsic motivation [45]. It is possible that positive emotions are the mediator between basic needs and intrinsic motivation. Humans are motivated to experience positive emotions. This might be because of the link to intrinsic motivation. Since we asked our interviewees for their emotions and they recalled the basic needs of the SDT, it seems likely that emotions had been felt prior to experiencing intrinsic motivation. Emotions are probably not only an aspect influencing intrinsic motivation, but also an outcome of satisfying basic needs, and probably occur before intrinsic motivation.

### 4.4. Open Questions

The strengths of this study are the systematic analysis and the heterogeneous choice of the sampling concerning sex, school levels, and extracurricular sport activities. The sample was equitable in regards to sex and school levels as well as the number of hours spent on participating in extra-curricular sport activities per week. The interviews have been highly standardized in terms of setting, interviewer, and interview structure. While the sample size (*n* = 12) seems small, there were enough participants to be able to achieve successful saturation and a deep exploration of the data, which might provide a framework for future qualitative approaches to this topic. In the present study, it is a limitation that the interview questions were not derived from current literature. Future research might use current literature to create the interview guideline and its questions to focus on the research gap. Another open question is—due to the restriction of collecting students’ names—which we are not able to prove the semantic meaning of words used by the interviewees.

### 4.5. Implications

This study sets implications for both future research and practical work for teachers in PE. Future research is supposed to add observational data to analyze behavioral effects of emotions in contrast to the individual representation of emotional experiences, as identified in this study. Additionally, quantitative studies should be carried out to test the established model of emotions in PE. This will help to focus more closely on the role of emotions, for example in motivational theories, and to be able to better explain the affective part of motivation. Further research on the mechanisms of basic needs and emotions are required in order to answer the question of which role do emotions play in the context of SDT in PE. This helps to better locate emotions in SDT and to better explain self-determined behavior in the context of secondary school PE.

For teachers, our results can contribute to knowledge of how emotional experiences can be triggered in students during secondary class PE. However, practical implications based on our research results are hypothetical. Therefore, it can be assumed that teachers can relate to the four identified triggers. They may focus on their students’ interests to contribute to the attractiveness of the task, better cooperation, and interaction in motor tasks. This might affect social belonging and strategies for setting individual goals to improve students’ perceptions of competence. Finally, teachers may use teaching methods supporting autonomy in order to meet the students’ needs for autonomy. Nevertheless, these practical implications are not analyzed with an empirical methodology. Thus, before integrating the given results into teachers’ work in PE, different pedagogical interventions have to be analyzed empirically.

## 5. Conclusions

A crucial part of PE is increasing young people’s commitment and decreasing their dropout rates in physical activities and sports in a lifelong perspective. In this context, understanding students’ emotional experiences is important. The present results show specific mechanisms that function as triggers. These are the attractiveness of the task, social belonging, perceived competence, and autonomy concerning emotional experience. These triggers are already known in various motivational theories, for example as basic needs to achieve intrinsic motivation [47]. In the context of PE, it has to be analyzed if teachers may provide, for example, activities that have relevance for the students, interactive activities, exercises differentiated according to the students’ abilities and vary PE topics after asking students for their ideas. Additionally, this explicit knowledge about students’ emotional experiences due to internal and external triggers may support teachers in elaborating students’ emotional competences. For example, teachers may sensitize students for their own emotional experiences by asking them to name their emotions after the teacher induced a trigger in PE. It is not only important that the identified triggers should be analyzed by pedagogical research to help PE teachers in terms of building a lifelong adherence to physical activity in their students, but also that they are built upon in further research aiming to integrate the role of emotions into already existing theories like the SDT. Future research should focus on the role of emotions for self-determined motivation, for example, to answer the question if emotions have a function as a signal to develop a self-determined motivation.
